# Human-centered design as a guide to intervention planning for non-communicable diseases: the BIGPIC study from Western Kenya

**DOI:** 10.1186/s12913-020-05199-1

**Published:** 2020-05-12

**Authors:** Claudia L. Leung, Mackenzie Naert, Benjamin Andama, Rae Dong, David Edelman, Carol Horowitz, Peninah Kiptoo, Simon Manyara, Winnie Matelong, Esther Matini, Violet Naanyu, Sarah Nyariki, Sonak Pastakia, Thomas Valente, Valentin Fuster, Gerald S. Bloomfield, Jemima Kamano, Rajesh Vedanthan

**Affiliations:** 1 Duke University Medical Center, 10 Duke Medicine Circle, Durham, NC 27710 USA; 2 Division of General Internal Medicine, Duke University School of Medicine, 200 Morris St. 3rd floor, Durham, NC 27701 USA; 3 Icahn School of Medicine at Mount Sinai, 1 Gustave L. Levy Pl, New York, NY 10029 USA; 4 Academic Model Providing Access to Healthcare (AMPATH), P.O. Box 4606, Eldoret, 30100 Kenya; 5 Department of Behavioral Sciences, School of Medicine, College of Health Science, Moi University College of Health Sciences, Eldoret, Kenya; 6 Purdue University, Purdue University College of Pharmacy, Purdue-Kenya Partnership, West Lafayette, IN, PO Box 5760, Eldoret, 30100 Kenya; 7 Department of Preventive Medicine, Keck School of Medicine, University of Southern California, Los Angeles, CA USA; 8 New York University Grossman School of Medicine, 180 Madison Avenue, 8th Floor, New York, NY 10016 USA

**Keywords:** Non-communicable diseases, Kenya, Human-centered design, Delivery of healthcare, Problem-solving, Microfinance

## Abstract

**Background:**

Non-communicable disease (NCD) care in Sub-Saharan Africa is challenging due to barriers including poverty and insufficient health system resources. Local culture and context can impact the success of interventions and should be integrated early in intervention design. Human-centered design (HCD) is a methodology that can be used to engage stakeholders in intervention design and evaluation to tailor-make interventions to meet their specific needs.

**Methods:**

We created a Design Team of health professionals, patients, microfinance officers, community health workers, and village leaders. Over 6 weeks, the Design Team utilized a four-step approach of synthesis, idea generation, prototyping, and creation to develop an integrated microfinance-group medical visit model for NCD. We tested the intervention with a 6-month pilot and conducted a feasibility evaluation using focus group discussions with pilot participants and community members.

**Results:**

Using human-centered design methodology, we designed a model for NCD delivery that consisted of microfinance coupled with monthly group medical visits led by a community health educator and a rural clinician. Benefits of the intervention included medication availability, financial resources, peer support, and reduced caregiver burden. Critical concerns elicited through iterative feedback informed subsequent modifications that resulted in an intervention model tailored to the local context.

**Conclusions:**

Contextualized interventions are important in settings with multiple barriers to care. We demonstrate the use of HCD to guide the development and evaluation of an innovative care delivery model for NCDs in rural Kenya. HCD can be used as a framework to engage local stakeholders to optimize intervention design and implementation. This approach can facilitate the development of contextually relevant interventions in other low-resource settings.

**Trial registration:**

Clinicaltrials.gov, NCT02501746, registration date: July 17, 2015.

## Background

Globally, non-communicable diseases (NCDs) are the most common cause of premature mortality [1, 2]. Associated with mortality and prolonged disability, NCDs have negative impacts at the individual, community, and societal level due to increased utilization of health services, as well as loss of income and decreased productivity [3, 4]. NCD incidence and outcome are closely linked to social, economic, and environmental factors, disproportionately impacting poor and vulnerable populations [5–7]. In Kenya and other low- and middle-income countries, NCDs are associated with a substantial household financial burden of care, which significantly impacts access to care [8].

In Kenya, multiple studies have demonstrated the positive impact of microfinance on poverty [9, 10], and a small but increasing number of studies have demonstrated the synergistic effect of integrating microfinance and health interventions [11]. Most interventions combined microfinance with health education and did not affect the more complex task of healthcare delivery [12]. The Village Savings and Loans Association (VSLA) model, on which the microfinance model in this paper is derived, has improved food security and strengthened household income indicators in Africa [13, 14]. In this model, participants save money together through buying shares and can access loans by borrowing against their savings. Interest is paid back to the group, and accumulated shares and interest are later shared out to all the group members [15]. Additional informal and formal group-based savings and credit models also exist in Kenya, including microcredit lending through institutions and local moneylenders, savings via investment in livestock, and the Rotating Savings and Credit Associations (ROSCA) model, commonly referred to as merry-go-round, in which members take turns receiving a pot of shared savings over a particular time period [16].

### The need for contextualized interventions

Lack of transport, poverty, and poor quality of care are known barriers to NCD care in western Kenya [17]. Skepticism regarding the health system, fear of stigma, and socio-economic fragility also contribute to low utilization of available healthcare resources [18, 19]. This complex milieu necessitates targeted solutions that address both the health and economic realities faced by this population. Rather than replicate existing interventions in high-resource settings, development of new interventions must be innovative, striving to provide high-quality care while accounting for resource constraints and contextual factors [5]. Ideally, a replicable intervention design and evaluation process should be used, allowing for the flexibility to develop a contextualized intervention while using a standardized process that can be applied in diverse settings.

Human-centered design (HCD) is a problem-solving approach that utilizes a series of iterative, often non-linear steps to tailor-make solutions for complex problems [20, 21]. While similar to other participatory research frameworks in its inclusion of end-user feedback, HCD differs in its endeavor towards empathy, a deep understanding of the motivations and desires that govern human behavior, as the inspiration and core of intervention development [20, 22]. In this approach, end-users are invited to partner in the design and evaluation process in order to better understand, meet, and even preempt their needs. In a low-resource, complex setting where the phenotypic manifestations of disease drivers may differ significantly from well-resourced settings, these key principles of HCD can be leveraged to optimize intervention development and implementation.

In this paper, we describe how we adapt a four-step HCD approach to guide the development of an integrated model of group care and microfinance for NCD care in rural Kenya. We use this case to describe the potential of utilizing a HCD approach to guide the development of complex interventions in a resource-limited setting.

## Methods

### Setting

The Academic Model Providing Access to Healthcare (AMPATH), initiated in 2001, is a partnership between Moi University College of Health Sciences, Moi Teaching and Referral Hospital, and a consortium of North American academic medical centers [23]. AMPATH established a system of HIV care in western Kenya and has since expanded its clinical scope to include population health and NCDs [24]. At the time that this study was conducted, the Chronic Disease Management program at AMPATH had enrolled over 2000 patients with diabetes and 40,000 patients with hypertension, who were being cared for at nine rural health centers and 30 rural dispensaries. The program dispatches clinicians to rural clinics monthly, which are otherwise staffed by nurses. This study was conducted as part of the Bridging Income Generation and Group Integrated Care (BIGPIC) study which aims to evaluate the combination of microfinance and group medical visits for cardiovascular risk reduction in the AMPATH catchment area across four counties in western Kenya [25].

### Intervention design

**Fig. 1:**
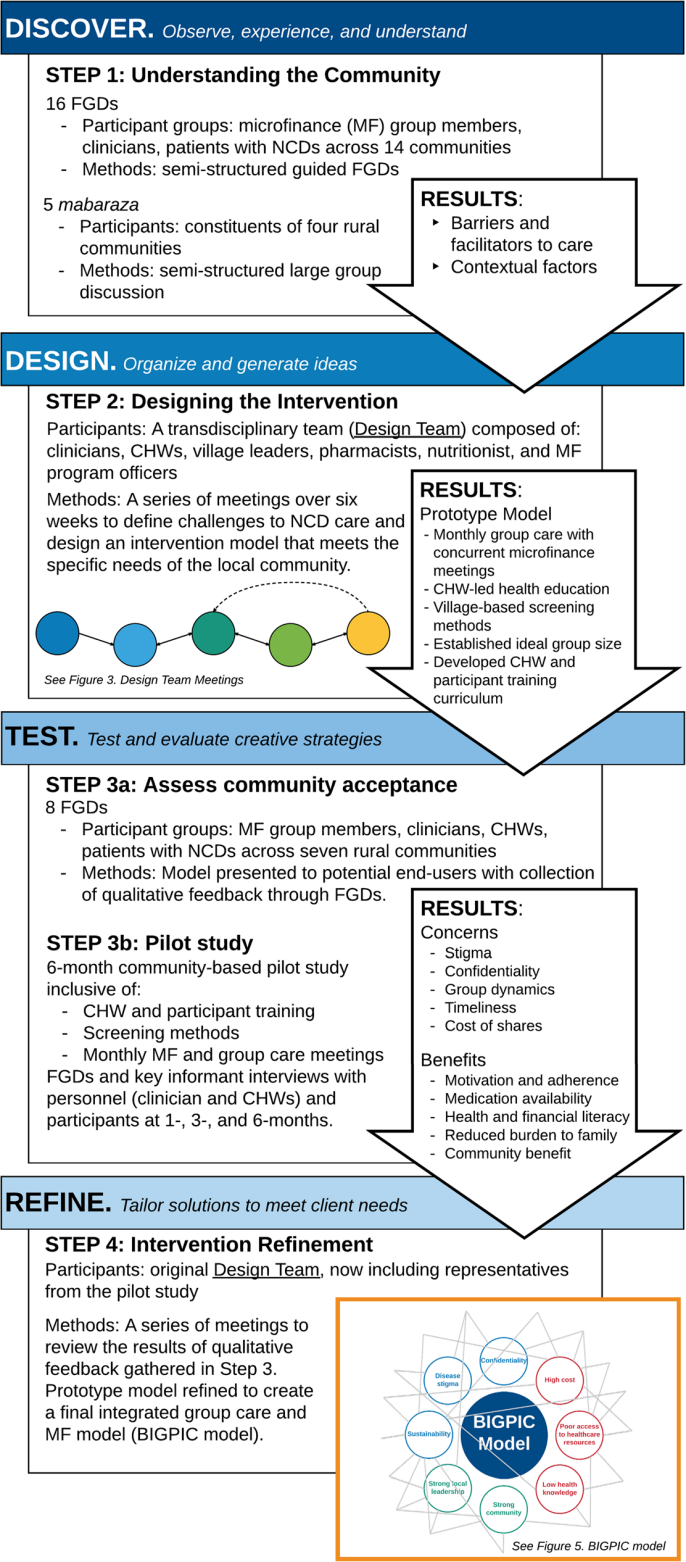
Human-centered design stages and activities in the BIGPIC design process. Steps 1–4 describe each stage of our project in the context of the HCD steps (Discover, Design, Test, and Refine). As HCD is an iterative process, the arrows describe how the results of each step impact the next

In this project, we adapted a pilot BIGPIC model that consisted of microfinance coupled with monthly group medical care visits with a rural clinician [26]. In this pilot model, participants with diabetes or hypertension are recruited to join the group and consist of at least 50% of group members. Group medical visits with a clinician occur immediately after each microfinance meeting. In order to further refine and adapt this approach to the local context, we utilized a HCD framework consisting of four steps – Discover, Design, Test, and Refine – with an emphasis on community and end-user engagement at each stage (Fig. 1). As HCD is an iterative process, the steps are described in sequence in the Methods and Results sections.

#### Step 1. DISCOVER: understanding the community

The first step in refining the BIGPIC model was to understand the strengths and needs of the local community. We utilized a combination of qualitative research methodologies to explore community and individual perspectives. The primary goals were to identify existing barriers to NCD care, and to identify contextual factors, barriers, and facilitators that could impact intervention design, implementation, and sustainability. We held *mabaraza,* traditional East African community gatherings, to discuss community perspectives in an open format. We also hosted focus group discussions (FGDs) consisting of 10–15 individuals with common characteristics (rural clinicians, community health workers, patients with NCDs, and microfinance group members) to explore individual perspectives. All qualitative studies were led by local team members utilizing a semi-structured guided interview in local languages.

#### Step 2. DESIGN: designing the intervention

##### Design team formation

**Fig. 2:**
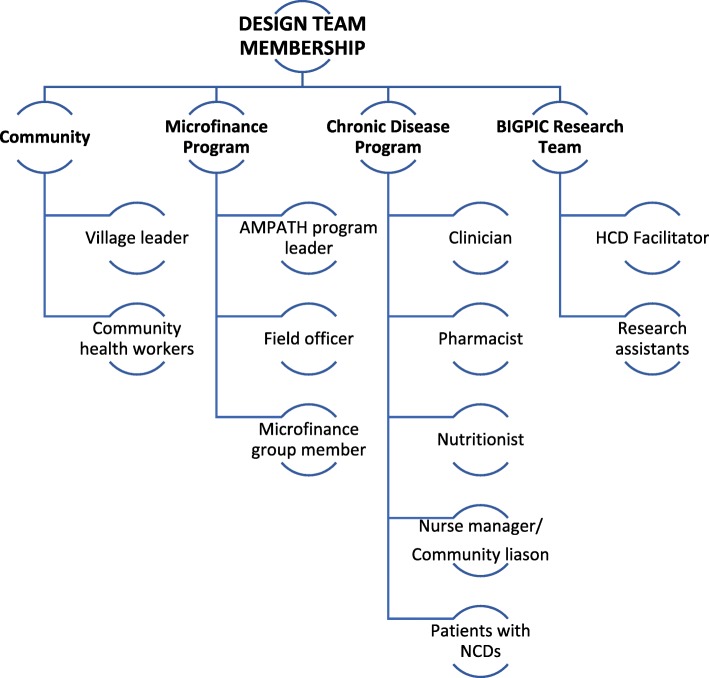
BIGPIC design team members

A transdisciplinary team of researchers and community stakeholders led the intervention design, implementation planning, and evaluation process (Fig. 2). The goals of the Design Team were to define the challenges to NCD care based on community input and design an intervention model to meet the needs and challenges of the end-users. Potential participants were identified through snowball sampling and local connections and invited to participate based on personal or professional experiences with NCDs or microfinance. All Design Team members represented end-users, those who may deliver or receive the intervention.

##### Design team meetings

Design Team meetings were conducted in a series of weekly three-hour meetings over 6 weeks, following objectives and activities adapted from HCD methodology [21]. An initial agenda was developed, however, timing for each step was flexible.

**Fig. 3:**
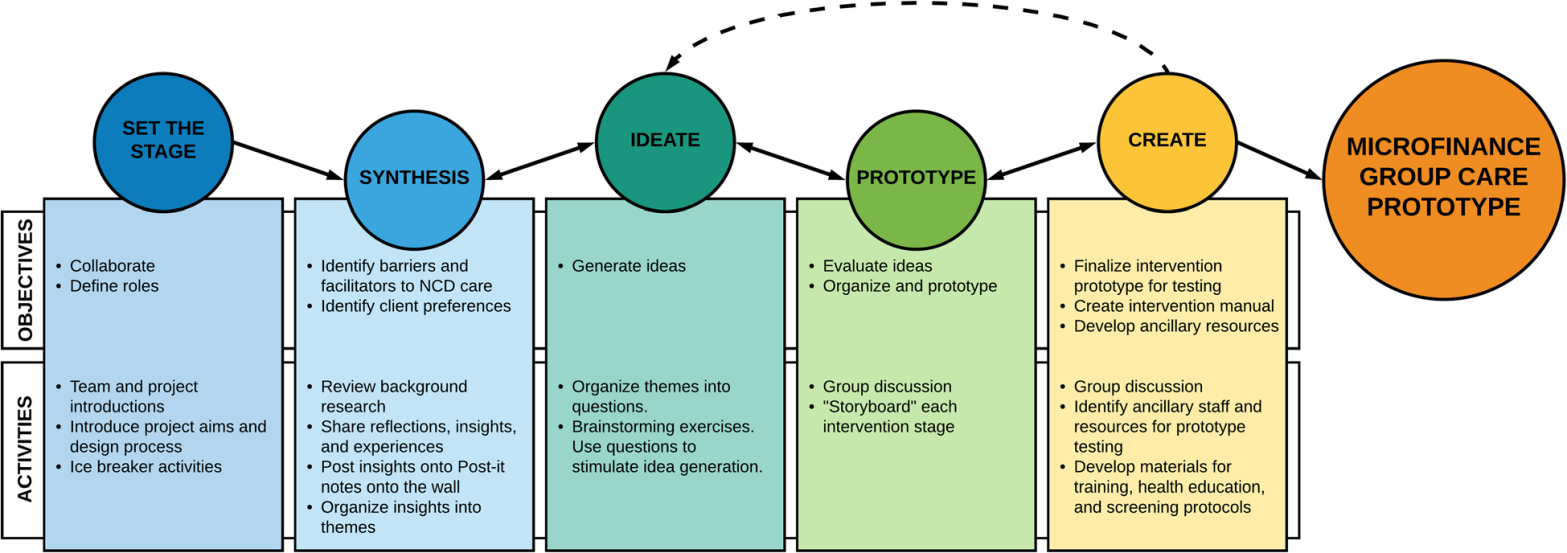
Format of BIGPIC design team meetings

The specific objectives and activities utilized in the Design Team meetings are described in Fig. 3. The forum and structure of the meetings were intentionally collaborative and interactive to encourage discussion and creativity. In Synthesis meetings, Design Team members reviewed qualitative data gathered in Step 1 and shared their insights, experiences, and questions to help engender a deep understanding of the strengths and barriers related to NCD care in western Kenya. Insights were posted visually and reorganized into broad themes which represented potential intervention barriers, facilitators, or unmet needs (Fig. 6**,** Table 1). Within each theme, questions were developed to facilitate brainstorming in Ideate meetings. For example, for the theme “Information/Engagement,” we asked, “how might we incorporate health education into patient care?” We learned in Step 1 that microfinance is generally considered a women’s activity which may preclude male participation, so for the theme, “Gender,” we asked, “how might we create a model that is responsive to the needs of men?” Each question was formed to catalyze group discussion to address important and nuanced aspects of the intervention that would ultimately impact intervention acceptability and sustainability.

**Table 1:** Key themes identified in Step 2, Synthesis meetings

Key Themes	
Information/engagement	
Gender	
Finance/Cost	
Attitude/Commitment	
Time	
Confidentiality	
Knowledge	

*Preliminary results from Step 1 presented to the Design Team were organized into Themes, along with member contributions of personal insights and experiences*

All ideas were noted and subsequently evaluated in group discussion for pros, cons, and feasibility in Prototype meetings. In the latter example above, we considered gender-specific groups, targeted screening locations when men tend to congregate, and community education, particularly through male leadership involvement.

Synthesis, brainstorming, and prototyping were cyclical and iterative steps that raised new questions influencing idea generation. In Create meetings, an initial prototype model was developed that combined group medical care and microfinance. Elements crucial to testing the prototype were developed in this step, including participant education materials, healthcare worker training curricula, and screening and evaluation protocols.

#### Step 3. TEST: assess community acceptance and pilot study

##### Acceptability studies

Qualitative feedback was gathered to assess community receptiveness to the proposed prototype model. FGDs of 10–15 individuals were conducted with groups of rural clinicians, microfinance group members, patients with NCDs, and CHWs. A question guide was used to steer the discussion in a semi-structured format. We performed a thematic analysis of the qualitative feedback utilizing NVivo.

##### Feasibility pilot study

Implementation strategies and the prototype, as defined in Step 2 Results, were piloted in one rural community in western Kenya. Adults who screened “positive” were those with elevated blood pressure or elevated fasting blood glucose. Inclusion criteria of those who screened positive were newly screened adults, previously screened adults who had never linked to care, or existing patients who had linked to care in the last 6 months. Those with diabetes or hypertension who did not meet the inclusion criteria were able to join the group as non-study participants to a maximum group size of 30 members. Participants and local CHWs subsequently received training in microfinance, hypertension, and diabetes. Qualitative feedback was elicited from the participating rural clinician, CHWs, and group participants at one, three, and 6 months using guided interviews and FGDs.

#### Step 4. REFINE: intervention refinement

The Design Team subsequently reconvened to evaluate the results of the acceptability studies and feasibility pilot study. In addition to the original team, representatives from among the pilot study participants were elected by their peers to take part in the reevaluation process. In a series of meetings, the Design Team reviewed the feedback and developed a final BIGPIC model.

## Results

### Step 1. DISCOVER: understanding the community

Five *mabaraza* and 16 focus group discussions were conducted across 11 sub-counties in western Kenya. Results from these qualitative studies indicated that cost, lack of medication availability, distance to health facilities, earned skepticism of the health system, socio-economic fragility, and stigma were significant barriers to accessing and maintaining NCD care [27].

### Step 2. DESIGN: designing the intervention

Our iterative design process resulted in an initial prototype model combining microfinance with monthly group medical care visits with a rural clinician. Compared to the original BIGPIC pilot model, this prototype engaged community health workers (CHWs) as group liaisons, included a CHW-led health education didactic at every meeting, and emphasized community-based recruitment approaches.

### Step 3. TEST: assess community acceptance and pilot study

Approximately 90 individuals in the community were screened, and 31 participants (12 male, 19 female) were enrolled to form the pilot study group. The group included a mix of different ethnic tribes and participants ranged in age from 36 to 75. In total, 17 FGDs and guided interviews were conducted (*N* = 110) at which point we achieved content saturation.

**Fig. 4:**
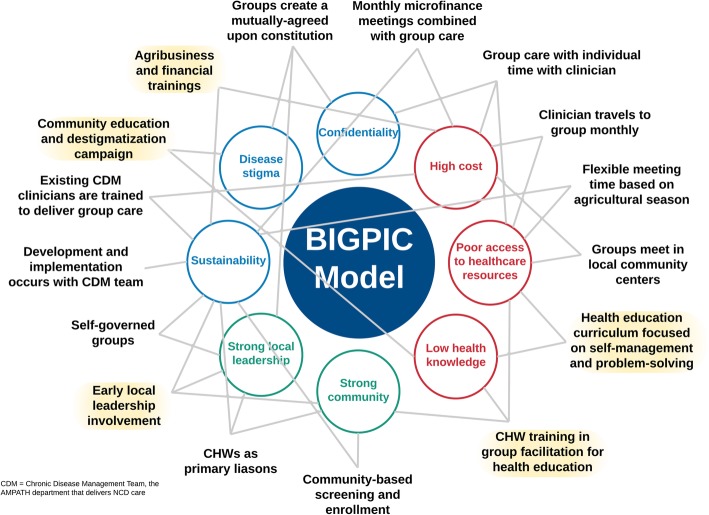
Benefits and Concerns related to the BIGPIC model

In general, the initial BIGPIC prototype model was found to be acceptable, with multiple perceived and anticipated benefits at the individual, family, and community levels (Fig. 4). Participants reported improved access to medical services by mitigating the need to travel, decreased cost of medications, peer support, and medication reliability as important benefits. Community level feedback provided additional insight to alleviate preexisting concerns regarding the prototype model, highlight persisting concerns, or raised new concerns not previously identified.

For example, one concern alleviated by the initial prototype regarded gender and group dynamics. In qualitative studies in Step 1 and Step 3, some community members expressed concern that differences in age and gender could affect group participation, given a strong culture of both gender and age-based hierarchy. Unintentionally, our pilot testing group was mixed not only in gender but also age and ethnic tribe. Yet, participants stated they found diversity in age, gender, and background to be a strength of the group, providing a sense of healthy competition between old and young, and allowing the younger members to take leadership roles and provide translation to their local language for the older members. Elected leaders in the group were both male and female, and gender differences did not negatively impact group dynamics.

New concerns elicited through this process included maximum group size and concern that this could lead to clinician burn out. Additionally, participants requested increased flexibility in scheduling monthly meetings so as not to interfere with their annual harvest schedule, and to expanded availability of commonly used medications. Researchers and CHWs noted low interest and engagement in the health education didactic sessions provided by the CHWs.

Persisting concerns shared by pilot participants included apprehension that participation would be limited by stigma associated with illness such as HIV. Participants emphasized the importance of anticipatory community education to enhance community receptiveness, and suggested strategies to facilitate this. Based on their prior experiences with brief lifecycles of programs due to limitations in funding and service delivery, participants and local leaders also expressed concern regarding the sustainability of the program, and noted these prior experiences may discourage some from joining. They also reported that income generally is low among their community and requested seed money or incentives to jump start their savings.

### Step 4. REFINE: intervention refinement

The final BIGPIC model consists of an integrated group care and microfinance model, with specific changes to the prototype model described in Table 3. These include expanded access to common medications, a reduction in maximum group size, and clarification of protocols with CHWs to coordinate changes in meeting times during the harvest season.

In response to the low interest in health education didactic sessions, our final model included a redesigned education curriculum, which shifted the focus from didactic sessions on NCD topics to strategies of shared learning. This included a group facilitation curriculum to help CHWs facilitate and guide discussions. For example, the CHW may choose a topic such as medication adherence, and guide a discussion of challenges to medication adherence, encouraging participants to share potential strategies to improve. Examples of other suggested topics include diet, exercise, oral hygiene, alcohol use, and stress management.

As suggested by local leaders and community members, our final implementation strategy included increased efforts towards community sensitization of our intervention and NCDs. We placed an increased emphasis on partnership with local community leaders, and provided reassurance that our BIGPIC model remains under Chronic Disease Management Team purview, a known and trusted presence in the community.

Finally, an extensive discussion took place regarding potential program incentives and seed money. Given community concerns for sustainability, the Design Team felt strongly that providing seed money for each newly formed group would be counterproductive as it would be dependent on grant funding. An intentional decision was made not to provide monetary incentives, but to instead scale up training in money management and agribusiness.

## Discussion

**Fig. 5:**
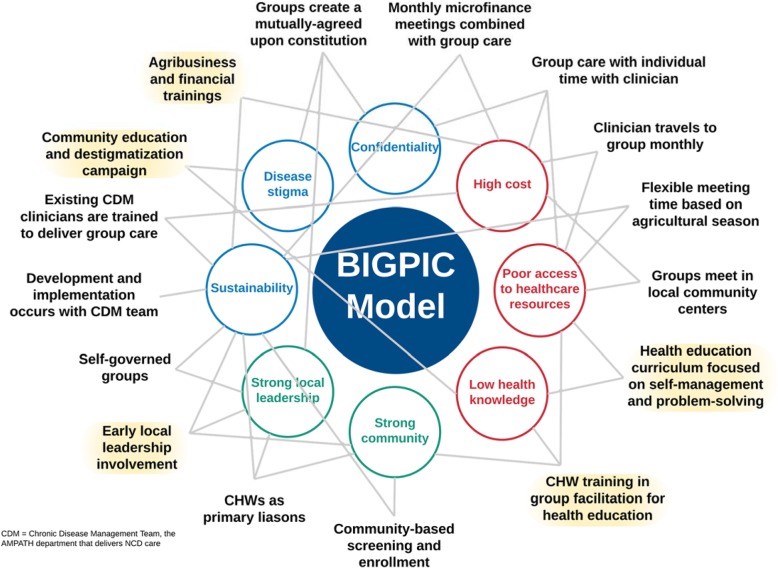
The BIGPIC model. The final BIGPIC intervention consists of an integrated group care and microfinance model. In this figure, the surrounding circles represent the unique milieu that has informed BIGPIC’s development. These include community strengths (green text), barriers to care (red text), and concerns regarding the BIGPIC model (blue text) elicited from community and pilot participant feedback, as described in Fig. 1 (Steps 1, 3, and 4). The surrounding descriptors in black text are key features and implementation strategies of the BIGPIC model. Each can be mapped to a community-driven strength, barrier, or concern. The text highlighted in yellow represents changes that were made during the Design Team Re-evaluation (Fig. 1, Step 4) in response to participant feedback

The refined BIGPIC model was developed using a four-step HCD framework, resulting in the development of a health care delivery model targeting health behaviors, medication adherence, and financial barriers to accessing healthcare in rural Kenya. We gathered insights and opinions from the community and formed a transdisciplinary Design Team of health professionals and community members to evaluate our data and create an initial prototype. This prototype was tested over six months and fine-tuned through community feedback to enhance acceptability and sustainability. The resulting BIGPIC model combines the benefits of microfinance with the peer support available through group medical care to enhance management of hypertension and diabetes. Key insights that developed through the HCD process informed both prototype features and implementation strategies and can be mapped directly to the strengths, needs, and concerns elicited from the community (Fig. 5**,** Tables 2-3). Currently, this product is the primary intervention of a four-arm randomized control trial to fully evaluate its impact [25]. Pending the final results of the randomized control trial, we are committed to working with stakeholders to scale up the model if it is found effective.

**Table 2:** Key insights and BIGPIC prototype features

KEY INSIGHTS	PROTOTYPE FEATURES	IMPLEMENTATION STRATEGY
*Strength:* Community, sense of brotherhood	Group-based care model to provide peer support and education Locally-based CHWs facilitate group formation	Community-based health screening to ensure local group formation
*Strength:* Community leadership	Participants elect group leaders and are self-governed by a mutually agreed upon constitution.	
*Barrier:* High cost of care (medications, transport, cost of services, caregiver burden)	Group care is combined with a microfinance program to increase individual access to funds for personal or medical use. Clinician brings basic medication supply box at every visit	Community-based health screening to ensure local group formation. Rural clinician and CHWs travel to group meetings at local community centers.
*Barrier:* Far distance to health facilities and poor quality roads	Community-based groups are linked with a local CHW.	Community-based health screening to ensure local group formation. Rural clinician and CHWs travel to group meetings at local community centers.
*Barrier:* Poor quality of existing physician-patient relationships	Same physicians return to the group as much as possible. Clinicians trained in group care are existing CDM clinicians.	
*Concern:* Variable group dynamics, particularly between age groups and gender	Participants create and sign a mutually agreed upon constitution that emphasizes self-governance and conflict resolution. Groups have a minimum number of study participants, and participants can bring additional friends/family to join the group until the maximum group size is attained.	
*Concern:* Stigma associated with illness or with AMPATH’s reputation as an organization for people with HIV.		Increased efforts for community education and destigmatization. Remove AMPATH logo from trucks.
*Concern:* Confidentiality	Group constitution includes a confidentiality clause that is created by the group members. Time is allotted for individual clinician assessment at every group care meeting.	
*Concern:* High cost of participation (share value) may prohibit some from joining	Group members agree upon share value at the start of the group. Limited number of shares can be bought per meeting.	
*Concern:* Sustainability of new programs	No external funding/seed money is required to start a microfinance group. Clinicians trained in group care are existing CDM employees.	Early local and governmental leadership involvement. Implementation occurs with existing CDM teams.

*Key insights elicited from the design process can be mapped directly to prototype features and implementation strategies. CDM - Chronic disease management*

**Table 3:** BIGPIC Re-evaluation changes

INITIAL PROTOTYPE FEATURE	FEEDBACK/CONCERNS	MODIFICATIONS
Monthly meeting time determined by clinician availability.	Participant availability may change based on agricultural season.	CHWs function as primary liaison with medical team to coordinate best meeting time before the end of each month.
Group education on NCDs at the time of group formation and before every monthly meeting.	There is low interest in group education.	Health education time is modified from didactic teaching to facilitated group discussions on self-management and problem solving. CHWs receive training in group facilitation.
Maximum group size of ~ 30 participants.	Large groups may overburden clinicians.	Maximum group size is decreased to ~ 20 participants.
Village-based health screenings to recruit intervention participants.	Concern for disease stigma may preclude willingness to join groups	Renew efforts to increase community health and intervention awareness. Remove AMPATH logo from clinician vehicles.
Clinician brings a toolkit of common medications for chronic disease management.	Availability of other commonly used medications (i.e., ibuprofen, antibiotics).	Toolkit of medications needed communicated to AMPATH pharmacy.
Community entry focused on local leadership.	Concerns regarding program sustainability.	Community entry and scale up includes multiple levels of leadership. Given CDM program is well known, emphasize roll out is in partnership with the existing CDM program. No seed money provided, but increased agribusiness and financial trainings.
Microfinance training during group enrollment, and CHW-led health education didactic sessions every month.	There is low income generation among community members, particularly elderly and those with low education levels.	Agribusiness and financial trainings are incorporated. Health education time is modified as above.

*Feedback and concerns elicited from pilot participant feedback informed key intervention modifications*

**Fig. 6:**
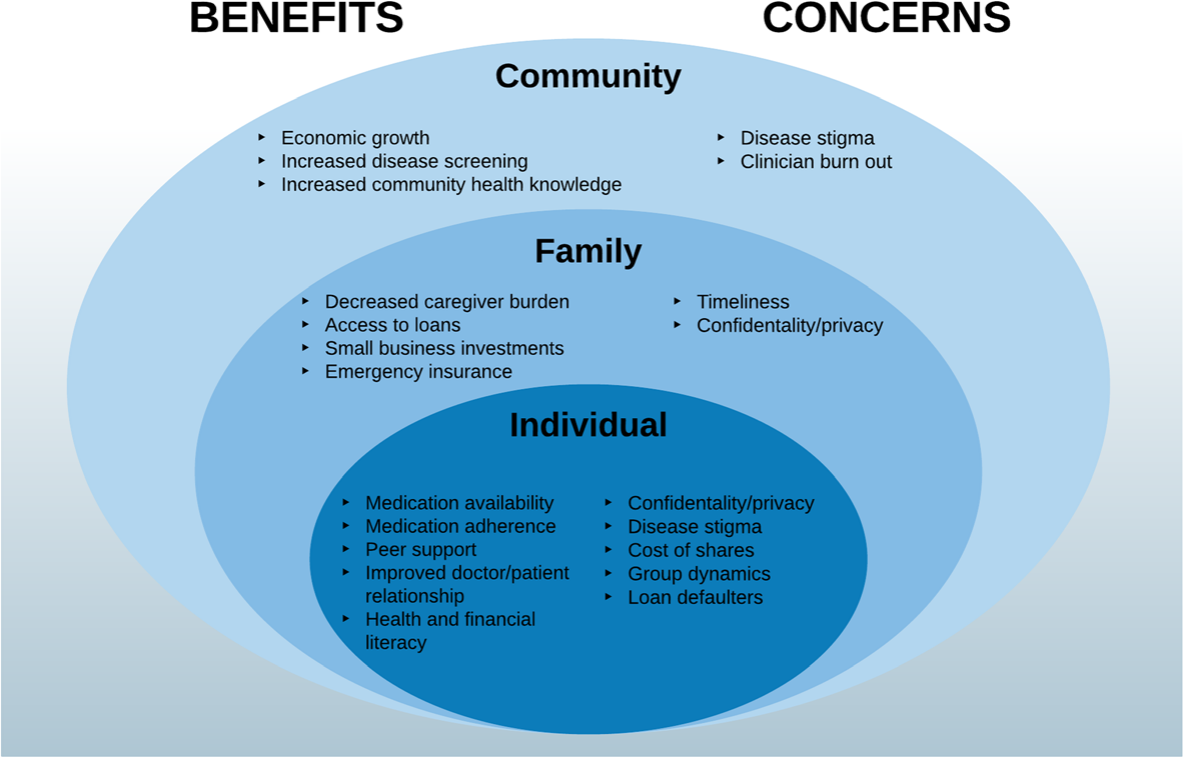
Key themes were organized together to stimulate idea generation

While the potential to leverage HCD for NCD care has been previously described [28], our study is one of the first to our knowledge to use HCD for a complex NCD intervention of this scale [12] . As described by the recent Lancet Taskforce on NCDs, poverty stems from and is exacerbated by the global burden of NCDs, and innovative means are needed to address the household economic burden of care in order to alleviate global poverty, decrease premature deaths, and progress towards the United Nations’ Sustainable Develop Goals in Kenya and other low- and middle-income countries [3, 29, 30]. Empathy-driven HCD at its core strives to understand the key drivers of human behavior and can be leveraged to help bridge the “knowing doing gap” that frequently characterizes poor adherence to prescribed lifestyle changes for NCD management such as dietary changes, weight management, and tobacco use [28, 31]. In limited resource settings, HCD is a process that can complement and support existing approaches to shaping NCD control such as the World Health Organization STEPwise approach [32]. HCD is one approach to optimize stakeholder engagement, and as in the example of BIGPIC, it can propel an understanding of local factors into the development of a contextualized intervention. In this study, our approach utilizing HCD for complex intervention design aligns with the Medical Research Council guidelines for developing complex interventions, utilizing a systematic approach to a development-evaluation-implementation process that is tailored to local circumstances [33]. Similar approaches to design-thinking have been described with other disease processes in low-resource settings [34–36].

Health inequities including those experienced by our catchment communities in Kenya are deeply rooted in complex social determinants, and increasingly need to be addressed in cross-sector and transdisciplinary partnerships [37]. Our HCD process provided a means to gather data and interact with the community to understand strengths and barriers to care, while also inviting community members to participate in innovation to enact changes in priority areas [38]. Through early involvement of stakeholders, we were able to not only address critical concerns early in the intervention design process, but also build partnerships with local stakeholders that would later be critical to the success of intervention implementation. Many components of our intervention and implementation strategies were illuminated through critical insights from end users throughout our HCD process. For example, multiple community members voiced concern for a sustainable intervention that would engage with local leaders and not be dependent on external funding. The use of an economic-based intervention as well as many of the features of this microfinance and group care model are in response to these lessons learned.

### Facilitators of success

Our intervention development operated on the backdrop of AMPATH’s existing partnerships with communities in its catchment areas. Its existing programs in NCD management and microfinance, network, and presence in rural communities helped to facilitate participant recruitment as well as engage with local leadership. AMPATH’s existing Chronic Disease Management program and resources including rural clinicians, clinical liaisons, and knowledge of the local communities allowed us to scale up our prototype more quickly and effectively. Increased availability of cellular service in rural areas made it possible for us to access patient records even in remote areas for continuity of care.

Notably, the Kenyan National Hospital Insurance Fund (NHIF) has recently extended its benefits to include NCD care in its benefits package. Our HCD process can provide insight as to the development and type of intervention that can be successfully incorporated under the benefits offered by NHIF.

### Limitations

Perhaps one of the greatest strengths of HCD and a vital lesson-learned in our design process is the importance of listening to and collaborating with our participants, which helped us to better understand their challenges and priorities. However, HCD can be a time-consuming process that may not be feasible for all project timelines and resources. Stakeholder buy-in and active engagement throughout the HCD cycle is essential, both at the personnel, institutional, and governmental level. This may be difficult to garner in some circumstances whether through lack of availability or lack of familiarity with this specific approach to intervention development.

Additionally, as use of HCD is still fairly novel in resource-limited settings, the presence of a facilitator familiar with the HCD process and tools is necessary but may be cost- and time–prohibitive, particularly if intervention development takes place over weeks to months. Similarly, while the formation of a multidisciplinary design team is a critical strength of HCD that begets a deeper understanding of the local context and paves the way for future intervention implementation, coordination of a 10 to 15 person team across diverse educational, language, and geographical backgrounds may be challenging**.** To combat this, HCD practitioners may consider shorter and faster cycles of prototyping for less complex interventions, in order to efficiently evaluate ideas and integrate lessons-learned for continuous improvement and sustainability. Beginning with shorter cycles may also help gain stakeholder support for subsequent longer cycles of more complex intervention development.

We also recognize that HCD is a process that requires tolerance of ambiguity, pivots, and prototyping—factors that can seem to be in opposition to traditional hypothesis-driven research methodologies [38]. However, we feel that HCD is a process for the design and development of interventions and implementation strategies that are both desirable and feasible in the local context, which can then be evaluated with traditional hypothesis-driven statistical methodologies. In our study, we have combined HCD with a more traditional randomized controlled implementation research trial to evaluate the effectiveness of the intervention [25].

Finally, there is growing enthusiasm in both academic medicine and global health spheres for social innovation and design thinking as tools that are more capable and responsive to the needs of end users [38–40]. However, there is still limited evidence regarding the impact of design thinking methodologies and related concepts on health outcomes [41]. Additional research is needed to evaluate the impact of participatory methodologies such as design thinking and social enterprises on health outcomes.

### Application to other contexts

The development and implementation of BIGPIC is one example of how HCD concepts can be used in resource-limited settings. Of particular relevance was the inclusion of transdisciplinary community stakeholders on our Design Team, who represented not only healthcare professionals, but also local community members, leaders, and microfinance experts. Our HCD process was inherently inclusive and collaborative, inviting innovation and feedback in every stage of development, and thrived through partnership and collaboration [42]. Its applications can be imagined broadly in both complex intervention development such as ours, or in more simple settings of adapting a known model or intervention to local context [38]. While it is unlikely that our exact HCD design and group care and microfinance model will be replicated wholesale in other contexts, our described process offers relevant lessons in low-resource settings both in the United States as well as abroad, in line with a scoping review of design thinking in global settings [38].

Recognizing that there are universal elements to care that are common across geopolitical and financial landscapes, we advocate for context-specific interventions that can help to optimize care in these settings. However, we recognize that potential unintended consequence is that such specificity may lead to variability and inequities in care. For this reason, we urge caution with planning for context-specific settings.

## Conclusions

In this study, we describe how a four-step HCD framework was used to tailor NCD service delivery to address multiple barriers to care for patients with hypertension and diabetes in western Kenya. HCD provided a means to engage early with local stakeholders, and the process of iteration and feedback was critical to address stakeholder concerns and optimize intervention design and implementation. While this approach to NCD intervention planning may be time-intensive, it resulted in an intervention package that is tailored to the local context and well-received by stakeholders. Future initiatives can use HCD to partner with local stakeholders to find innovative ways to address complex health problems in resource-constrained settings.

## Data Availability

This study complies with the NIH Public Access Policy, which ensures that the public has access to the published results of NIH funded research, and therefore, all results have been (and will be made) available from final peer-reviewed journal manuscripts (including this one) via the digital archive PubMed Central upon acceptance for publication.

## Abbreviations

AMPATH: Academic Model Providing Access to Healthcare
BIGPIC: Bridging Income Generation with Group Integrated Care
CARE: Cooperative for Assistance and Relief Everywhere
CDM: Chronic disease management
CHW: Community health worker
FGDs: Focus group discussions
HCD: Human-centered design
MF: Microfinance
NCD: Non-communicable disease
VSLA: Village Savings and Loan Association
NHIF: National Hospital Insurance Fund
