# Correction to: Human-centered design as a guide to intervention planning for non-communicable diseases: the BIGPIC study from Western Kenya

**DOI:** 10.1186/s12913-020-05345-9

**Published:** 2020-08-12

**Authors:** Claudia L. Leung, Mackenzie Naert, Benjamin Andama, Rae Dong, David Edelman, Carol Horowitz, Peninah Kiptoo, Simon Manyara, Winnie Matelong, Esther Matini, Violet Naanyu, Sarah Nyariki, Sonak Pastakia, Thomas Valente, Valentin Fuster, Gerald S. Bloomfield, Jemima Kamano, Rajesh Vedanthan

**Affiliations:** 1 Duke University Medical Center, 10 Duke Medicine Circle, Durham, NC 27710 USA; 2 Division of General Internal Medicine, Duke University School of Medicine, 200 Morris St. 3rd floor, Durham, NC 27701 USA; 3 Icahn School of Medicine at Mount Sinai, 1 Gustave L. Levy Pl, New York, NY 10029 USA; 4 Academic Model Providing Access to Healthcare (AMPATH), P.O. Box 4606, Eldoret, 30100 Kenya; 5 Department of Behavioral Sciences, School of Medicine, College of Health Science, Moi University College of Health Sciences, Eldoret, Kenya; 6 Purdue University, Purdue University College of Pharmacy, Purdue-Kenya Partnership, West Lafayette, IN, PO Box 5760, Eldoret, 30100 Kenya; 7 Department of Preventive Medicine, Keck School of Medicine, University of Southern California, Los Angeles, CA USA

**Correction to: BMC Health Serv Res (2020) 20:415**

**https://doi.org/10.1186/s12913-020-05199-1**

Following the publication of the original article [1], it was noted that due to a typesetting error the Figs. 1, 4, 5 and 6 are not correct.

**Fig. 1:**
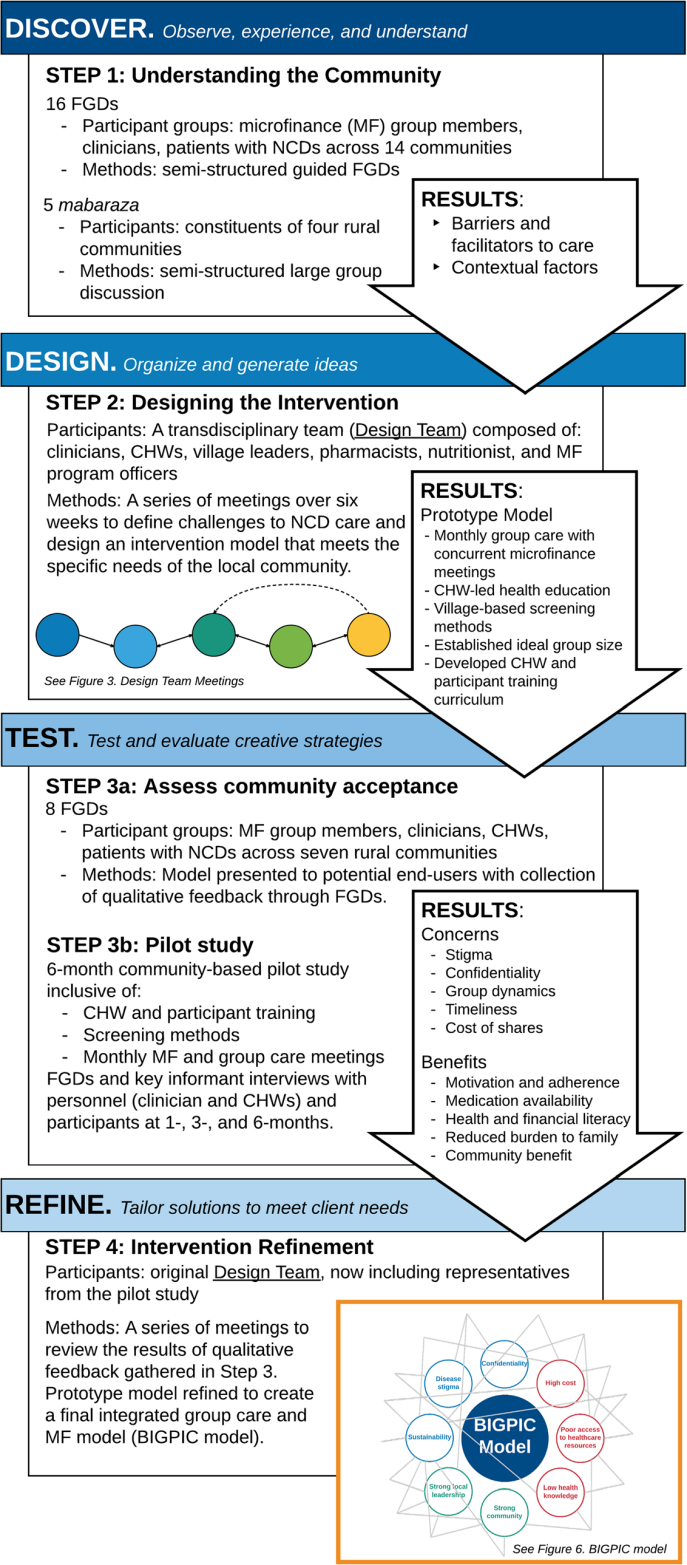
Human-centered design stages and activities in the BIGPIC design process. Steps 1–4 describe each stage of our project in the context of the HCD steps (Discover, Design, Test, and Refine). As HCD is an iterative process, the arrows describe how the results of each step impact the next

**Fig. 4:**
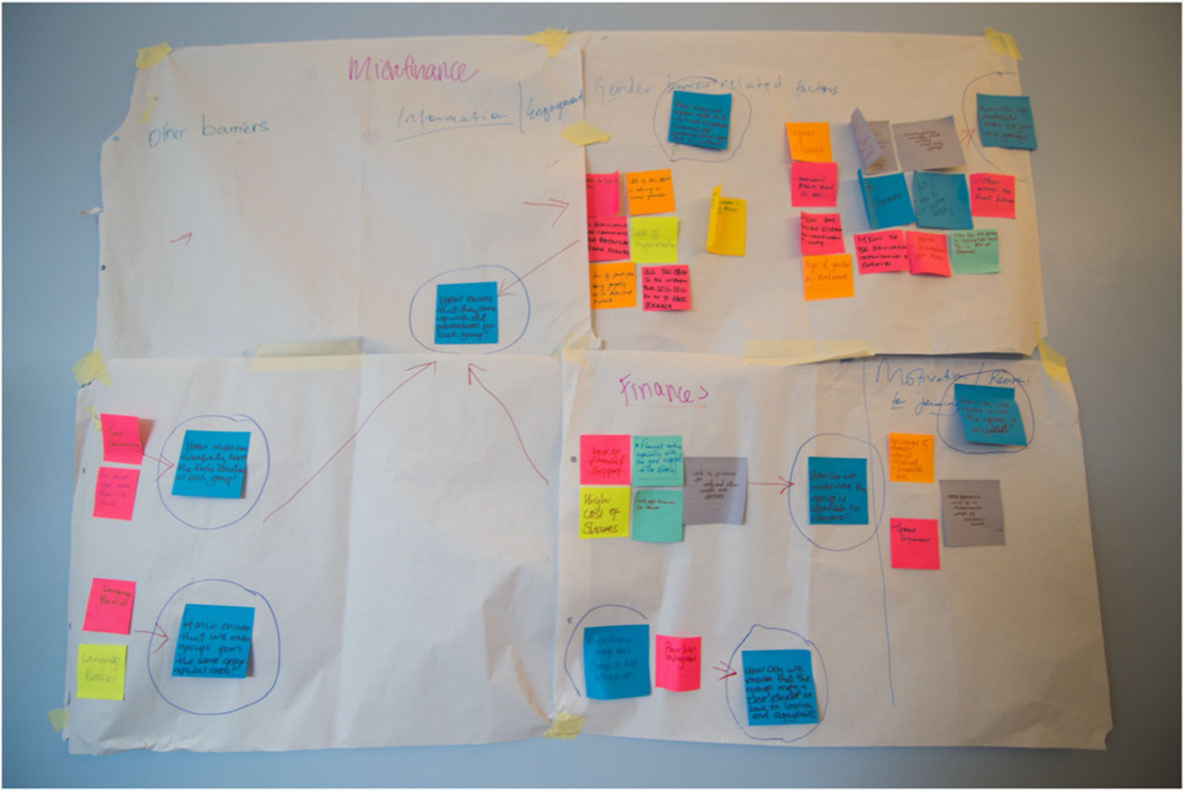
Key themes were organized together to stimulate idea generation

**Fig. 5:**
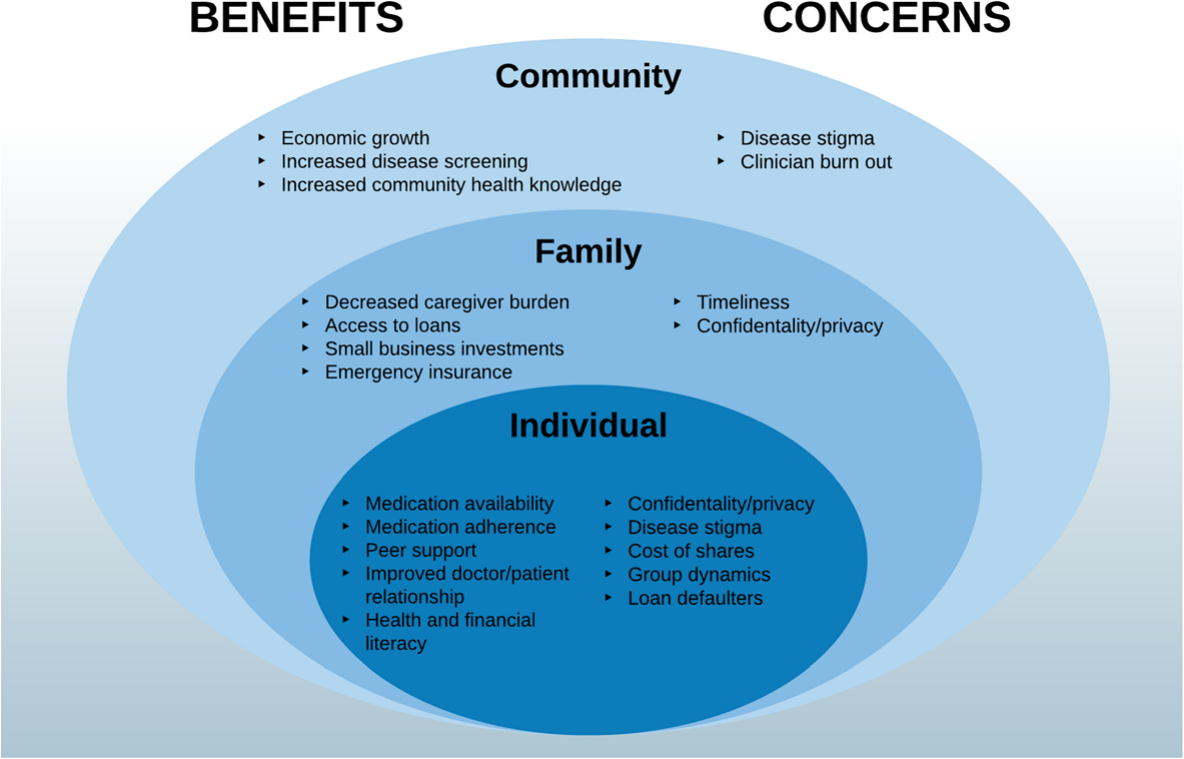
Benefits and Concerns related to the BIGPIC model

**Fig. 6:**
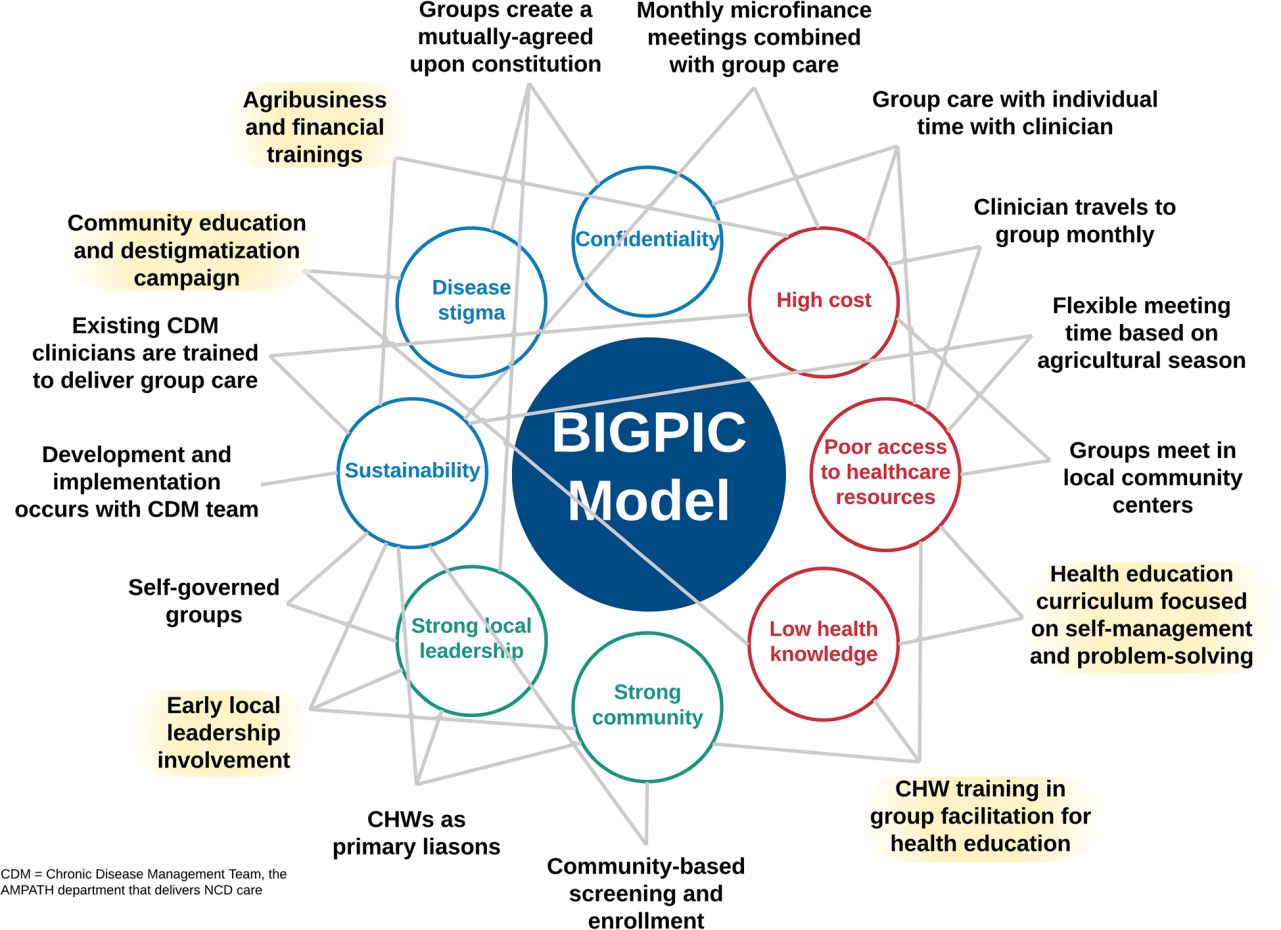
The BIGPIC model. The final BIGPIC intervention consists of an integrated group care and microfinance model. In this figure, the surrounding circles represent the unique milieu that has informed BIGPIC’s development. These include community strengths (green text), barriers to care (red text), and concerns regarding the BIGPIC model (blue text) elicited from community and pilot participant feedback, as described in Fig. 1 (Steps 1, 3, and 4). The surrounding descriptors in black text are key features and implementation strategies of the BIGPIC model. Each can be mapped to a community-driven strength, barrier, or concern. The text highlighted in yellow represents changes that were made during the Design Team Re-evaluation (Fig. 1, Step 4) in response to participant feedback

The correct figures have been included in this correction, and the original article has been corrected.

The author affiliations need to be revised as below and the original article has been corrected.

Claudia L. Leung^1^, Mackenzie Naert^2^, Benjamin Andama^3^, Rae Dong^2^, David Edelman^1^, Carol Horowitz^2^, Peninah Kiptoo^3^, Simon Manyara^3^, Winnie Matelong^3^, Esther Matini^3^, Violet Naanyu^4^, Sarah Nyariki^3^, Sonak Pastakia^5^, Thomas Valente^6^, Valentin Fuster^2^, Gerald S. Bloomfield^1^, Jemima Kamano^4^, and Rajesh Vedanthan^7*^
